# Retraction: The Role of Allograft Inflammatory Factor-1 in the Effects of Experimental Diabetes on B Cell Functions in the Heart

**DOI:** 10.3389/fcvm.2021.804542

**Published:** 2021-11-12

**Authors:** Frontiers Editorial Office

Following publication, concerns were raised to the editorial office regarding the reliability of the scientific evidence as presented in some figures. The authors failed to provide a satisfactory explanation during the investigation, which was conducted in accordance with Frontiers' policies.

This retraction was approved by the Chief Editors of Frontiers in Cardiovascular Medicine and the Chief Executive Editor of Frontiers. The authors did not agree to this retraction.

